# A Qualitative Exploration of General Practitioners’ Treatment Decision-Making for Depressive Symptoms

**DOI:** 10.1177/0272989X231166009

**Published:** 2023-04-14

**Authors:** Alex Stallman, Nicola Sheeran, Mark Boschen

**Affiliations:** School of Applied Psychology, Griffith University, Brisbane, Australia; School of Applied Psychology, Griffith University, Brisbane, Australia; School of Applied Psychology, Griffith University, Brisbane, Australia

**Keywords:** primary care, mental health care, depression, antidepressants, psychologist

## Abstract

**Background:**

General practitioners (GPs) provide the most antidepressant prescriptions and psychologist referrals in Australia, yet little is known about how they decide between treatments for depressive symptoms.

**Aims:**

This study examined the decision cues that GPs use when deciding how to treat depressive symptoms and the meaning they attribute to these associations.

**Methods:**

Structured interviews were conducted with 16 Australian GPs in a “think-aloud” verbal protocol analysis format. The transcripts were analyzed using content coding and thematic analysis, informed by the dual processes model of decision making.

**Results:**

Participants associated prescribing antidepressants with severe depressive symptoms, and psychologist referrals were the preferred initial treatment provided patients were willing to engage. Four main themes emerged from the thematic analysis: 1) psychologist as default, 2) the risk is just too high, 3) medication as supplement, and 4) drivers of antidepressants. Contrary to previous findings, participants identified a strong heuristic association between depressive symptoms and psychologist referral. Antidepressant prescription was associated with severe symptoms, higher risk, and a cluster of cues that lead them away from psychologist referral.

**Conclusions:**

Participants demonstrated an oversensitivity to depressive symptom severity, associating psychologist referrals with subclinical depressive symptoms, and starting antidepressants for suicidal ideation and significant functional decline.

**Highlights:**

- Psychologist referrals were the default preference for GPs when treating depressive symptoms, whereas antidepressants were reserved for patients with elevated risk from severe depressive symptoms and suicidal ideation.
- Participants’ conceptualization of depression severity was oversensitive compared with treatment guidelines.
- Contrary to treatment guidelines, GPs in this study demonstrated their discomfort for referring patients with suicidal ideation to a psychologist without initiating antidepressants.
- GPs should consider whether patients within their mild to moderate assessment range can be treated exclusively with more cost-effective means than psychological therapy such as e-mental health programs, guided self-help, and lifestyle changes alone.

General practitioners (GPs) in Australia occupy a crucial role in public mental health care. In 2021, 70% of GPs reported mental health as the most common health issue they treated,^1^ and 1 in 10 primary care patients present with depressive symptoms.^2^ Despite GPs’ ubiquitous position within mental health care, little is known about their treatment decision making for patients’ mental health needs. In Australia, GPs can provide a mental health care plan to patients with diagnosed major depressive disorder (MDD) that allows up to 10 government-subsidized psychology sessions per year.^3^ GPs in Australia receive little formalized training in treating depression throughout medical school or GP registrar programs. Most formalized mental health training occurs in acute hospital settings that do not encapsulate the subclinical depressive symptoms commonly seen in general practice, leaving the responsibility of education to GPs’ for ongoing professional development in mental health.^4^ Due to individualized rather than generalized education, we know very little about how GPs’ practice aligns with evidence-based practice nor how evidence-based practices are implemented into the complexities of primary care.

Australian treatment guidelines state that depression of all severities can be effectively treated with psychological therapy or healthy lifestyle changes and that antidepressants should be reserved for when this is not effective.^2^ GPs therefore have a choice of 3 broad treatment categories for depressive symptoms: prescribing medication, providing psychological and lifestyle interventions themselves, and referring to a specialist (i.e., psychologist). Data from Bettering the Evaluation and Care of Health survey found that 27.8% of total Medicare consults for mental health resulted in antidepressant prescriptions, while only 9.8% resulted in referral to a psychologist.^5^ Antidepressant prescriptions in Australia and other countries have been increasing for decades.^6^ However, a growing body of research has challenged the hypothesis that depression stems from depleted serotonin^7^ and highlights several negative long-term consequences of taking antidepressants such as libido reduction, dry mouth, weight gain, and withdrawal symptoms upon cessation.^8,9^

When asked about their approach to treating mental health issues, GPs report striving to achieve the “right care fit” for patients’ depressive symptoms using a variety of pharmacologic and psychological treatment options.^10^ GPs in Australia and internationally report using patient-centered decision-making processes to explore treatment options for depressed patients.^10–12^ In a large sample of New Zealand GPs, 84% explored alternative options to antidepressants with their patients before writing a prescription.^13^ However, it is unclear what specific information GPs attend to, elicit, and prioritize when deciding what treatments to recommend.

## Dual Processes Model of Decision Making

The dual processes model of decision making has been used to describe and inform clinical decision making in primary care.^14^ Two decision-making processes are described by the model: heuristic decisions are rapidly generated, automatic decision processes that rely on past experiences and limited cognitive analysis; systematic decisions are more slowly considered, rationalized, and logic driven.^15^ According to the dual processes model, decisions are made by collecting decision cues (pieces of information) and integrating them using 1 of the 2 decision processes, resulting in a decision outcome.^15^ Medical decision cues typically manifest as patients’ symptoms and their demographic characteristics.^14,15^

Certain symptoms and characteristics have been correlated with antidepressant prescriptions or psychologist referrals. A study using written patient vignettes with British GPs found that longer duration symptoms predicted antidepressant prescriptions.^16^ Patients’ gender, treatment preference, suicidal ideation, and impaired concentration also significantly influenced GPs’ decisions to prescribe antidepressants.^16^ Australian studies investigating GPs’ treatment of depression found that females and younger patients (16–29 years) were more likely to receive psychologist referrals.^17,18^ Patients older than 30 years and people of lower socioeconomic status were significantly more likely to receive antidepressant prescriptions.^17,18^ While GPs’ treatment decisions have been predicted by demographic characteristics and depressive symptoms, the decision processes used to make treatment decisions is poorly understood. This study will determine whether GPs’ decision processes for treating depression are aligned with treatment recommendations and reveal how GPs are allocating mental health resources at the primary care level.

This study examined the decision cues GPs attend to when deciding on treatment for depressive symptoms and how these cues are associated with the most common treatments. A mixed-methods study using content coding and thematic analysis was undertaken. More specifically, we addressed the following research questions:

RQ 1: Which decision cues do GPs associate with treatment options for depressive symptoms?
RQ 2: What meaning do GPs attribute to these associations when reaching a treatment decision?

## Method

### Participants

Seventeen GPs working across a variety of states and localities in Australia participated in the study, though one was excluded before the interview took place, as they did not prescribe antidepressants, leaving a final sample of 16 (see Table 1). By choosing to not prescribe antidepressants as a general rule, this participant did not engage in the more complex decision-making process used by other participants. The remaining GPs were in current practice and accepting of both antidepressants and psychologist referrals for treating depressive symptoms. Recruitment continued until there was both saturation of the data, whereby no new cues were being identified, and we had detailed descriptions of decision processes from a range of GPs.^19^

**Table 1:** Participant Demographics

Demographic	Value
Male	8
Female	8
Age, y	*m* = 48.1 (*s* = 14.1), *r* = 29–70
Years of experience	*m* = 17.1 (*s* = 16.1), *r* = <1–48
Practicing in QLD	12
Practicing in NSW	1
Practicing in WA	2
Practicing in VIC	1
Urban locality	12
Regional locality	4
Australian medical training	14
Irish medical training	2
Co-location with MH professional	9
No co-located MH professional	7
Total hours of MH training completed	*m* = 31.1 (*s* = 27.1), *r* = 5–100
5–15	7
20–30	4
>50	4
Missing	1
Very confident treating depression	6
Confident treating depression	10

NSW, New South Wales; QLD, Queensland; *r*, ran; VIC, Victoria; WA, Western Australia.

### Procedure and Materials

Participants were recruited via e-mails to practices and advertisements on Facebook and professional newsletters (e.g., RACGP) between April and November 2020 and could enter a prize draw for a $250 restaurant voucher as an incentive. Structured interviews were conducted via Zoom or telephone and were approximately 30 min in length. We used a verbal protocol analysis (VPA) “think-aloud” method of interviewing, a well-recognized structured interview technique for capturing ongoing or recently completed decision processes.^20,21^ VPA elicits actual thoughts, not explanatory or descriptive responses,^20^ and avoids imposing external structure on the participant’s own decision-making structure (see Appendix). VPA has been used previously in health contexts to examine information relevant to clinical judgments about patients.^22^ Interviews were digitally recorded and then anonymized and transcribed verbatim before being checked for accuracy against recordings. Transcriptions were then entered into NVivo 12 for coding. Ethics approval for the study was granted from Griffith University human research ethics committee (ref No. 2020/290), and all participants consented to their answers being included in research.

### Analysis

Data were analyzed using a mixed qualitative approach of content coding^23^ and latent thematic analysis.^24^ Content coding analysis was used to identify the decision cues described by participants, to calculate cue frequency, and categorize cues into their associated treatment option. Latent thematic analysis was informed by the dual processes model of decision making and conducted within an essentialist epistemological framework^24^ whereby it was assumed that participants’ responses accurately reflected their conceptualization of their decision processes. A single decision-making model was generated to encapsulate the processes described by all 16 participants.^25^ Responses that conflicted with the decision model were acknowledged during analysis but were not integrated into themes. Resulting themes were categorized into heuristic or systematic decision processes.

#### Content coding

The first author content coded 3 interviews initially to develop a coding protocol for decision cues and then categorized them into the associated treatment options. The emergent patient cues for the remaining 13 participants were then coded using this protocol, and each cue was grouped within the associated treatment category. Slight semantic changes were made to the coding protocol as coding continued and discussions occurred between authors. Twenty percent of responses were independently coded by a second coder to ensure accuracy and consistency (κ = 0.883). Frequency of cues were then calculated.

#### Thematic analysis

Latent thematic analysis was conducted in accordance with the procedures outlined by Braun and Clarke^24^ and using the dual processes model of decision making to guide conceptualization of patient information into decision cues and decision cues into decision processes. Associations between patient cues and treatments were framed as either heuristic or systematic decision processes. The first stage of coding involved identifying the most meaningful cues from content coding for each treatment option. A combination of cue frequency, surrounding context, and relevance to the research questions was used to identify initial themes. Stage 2 of the analysis involved a greater focus on the context surrounding each of the most prominent cues and the latent understanding of meaning beyond semantic descriptions of cues. In stage 2, a model was formed outlining the different decision pathways that led participants toward each treatment option for depressive symptoms.

Of note, all participants asked for clarification on the questions relating to the treatment option “GP management” and expressed confusion. Responses provided were vague and nondescript, so data pertaining to this treatment option were dropped from subsequent analysis.

## Results

Participants identified a total of 47 cues related to treating depressive symptoms. Table 2 displays the cues that participants associated with each treatment option, how many participants identified each cue, and whether the association was positive or negative. Psychologist referrals were identified as the default treatment preference and associated with patient willingness, whereas antidepressants were associated most frequently with severe depressive symptoms and suicidal ideation. Recommending both treatments simultaneously was associated with severe depressive symptoms.

**Table 2:** Cues Associated with Treatments for Depressive Symptoms by Participants

Treatment	Cue	No. of Participants	Association with Treatment
Antidepressants	Severity: severe	12	Positive
	Past success with antidepressants	12	Positive
	Patient willingness to take antidepressants	11	Positive
	Suicidal ideation	8	Positive
	Antidepressant family history	8	Positive
	Insomnia	8	Positive
	Age: older	7	Positive
	Age: middle aged	7	Positive
	Lack of progress with psychologist	7	Positive
	Physical comorbidities	6	Positive
	Gender: male	4	Positive
	Comorbid anxiety	4	Positive
	Duration of symptoms: long	4	Positive
	Past success with a psychologist	12	Negative
	Patient willingness to see a psychologist	11	Negative
	Age: younger	7	Negative
Psychologist referral	Patient willingness to see a psychologist	13	Positive
	Psychologist as default option	9	Positive
	Good insight	7	Positive
	Past success with a psychologist	7	Positive
	Age: younger	7	Positive
	Trauma history	5	Positive
	Gender: female	4	Positive
	Severity: mild to moderate	3	Positive
	Duration of symptoms: short	3	Positive
	Bad experience with a psychologist	13	Negative
	Past success with antidepressants	12	Negative
	Patient willingness to take antidepressants	11	Negative
	Poor access to a psychologist	9	Negative
	Poor insight	7	Negative
Combined	Severity: severe	12	Positive
	Lack of progress	6	Positive
	Past success	6	Positive
	Patient willingness to try both	6	Positive
	Duration of symptoms: long	5	Positive
	Insomnia	5	Positive
	Psychologist recommendation	5	Positive
	Patient unwilling to try either	6	Negative

Combined, simultaneous prescription of antidepressants and psychologist referral; Negative, decreased likelihood of choosing treatment; Positive, increased likelihood of choosing treatment.

### Themes

We identified 4 themes including 3 heuristic and 1 systematic process used by participants to associate cues with treatments and the meaning attached to these associations. The 3 themes capturing heuristic processes include: “Psychologist as a default treatment,” “The risk is just too high,” and “Medication as a supplement,” while a more systematic process is outlined in “Drivers of antidepressants.” We then present a visual summary of these 4 themes as a general decision-making process for treating depressive symptoms.

### Psychologist as a Default Treatment Option

**Figure 1:**
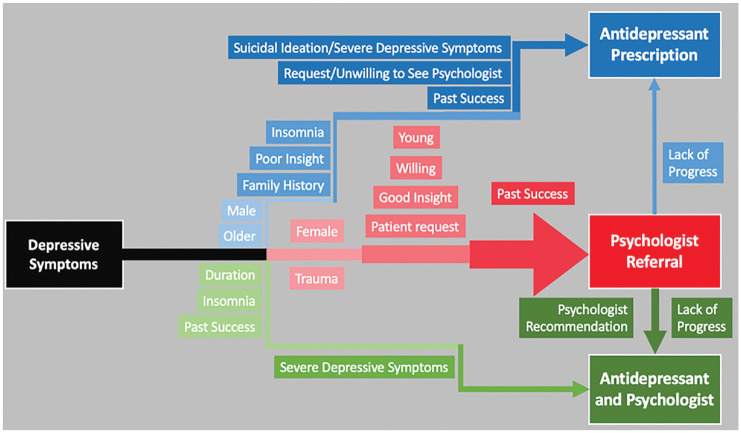
Visual model of general practitioner decision making for depressive symptoms. Psychologist referral was the default treatment option, with a range of cues that moved decisions toward prescribing antidepressants (either as the primary treatment option or in combination with psychological intervention). Cues with darker coloring and that are closer to the treatment indicate more frequent mentions.

Most participants described psychologist referrals as their preferred treatment for depressive symptoms, provided the patient was willing and able to engage, thus describing their first heuristic process (see Figure 1). For example, “I have a very low threshold for referring to a psychologist” (P06). When asked who would benefit from a psychologist for depressive symptoms, one participant stated, “Everyone. All my patients” (P11). Many participants also reported exploring lifestyle interventions alongside or before referring to a psychologist and being guided by patients’ willingness to engage with psychologists or take antidepressants (see Table 2). There were fewer and more generic cues associated with psychologist referrals compared with multiple specific cues associated with prescribing antidepressants, indicating fewer barriers for psychologist referrals than antidepressant prescriptions. For example, “I try and encourage probably most people, and that would be my first line before medication” (P10). Many of the cues associated with antidepressants were in fact barriers to psychologist referrals, such as patient preference and previous negative experiences with psychologists. Participants commonly associated lack of progress from therapy with prescribing antidepressants but did not associate a lack of progress from antidepressants with a psychologist referral.

One participant contradicted this theme with their decision framework. They preferred to initiate treatment with antidepressants or lifestyle management for most patients, adding a psychologist when this was not having the desired effect. For example, “Someone who . . . might be willing to take an antidepressant, but they’re actually quite persistent in negativity, and resistant to change, I might refer them to a psychologist” (P15).

### The Risk Is Just Too High

Severe depressive symptoms were cited as an exception to participants’ default preference of beginning treatment with a psychologist referral, preferring instead to stay with antidepressants, thus describing another heuristic association. For example, when asked what they have thought about when prescribing antidepressants, “…certainly the severity of the symptoms, things like if they’re just not, not able to concentrate, you know, enough to go to work, if their sleep pattern is severely disrupted.” (P13). Participants often referenced patient risk, either of suicide or a significant decline in their daily functioning when associating symptom severity with antidepressants. For example, “I don’t push people to take antidepressants unless they’re actually suicidal” (P14). When patient risk was elevated by suicidal ideation or sudden functional decline, participants preferred to start treatment with antidepressants before involving a psychologist. Once the medication had taken effect and patients’ functioning improved, participants might then refer them to a psychologist, “But you often do start them on antidepressants because I think they need those to get to a point where they can do counselling” (P01).

### Medication as a Supplement

Another function of antidepressants described by participants was to improve the functioning of patients who are seeing a psychologist but struggling to engage in therapy. For example, “it’s usually when people aren’t really responding to psychology . . . I tell them that . . . the antidepressant will kind of give them the space to interact with therapy” (P03). Many participants stated they often prescribe antidepressants at the psychologist’s recommendation based on a lack of progress in therapy. Participants stated in these circumstances that antidepressants were prescribed to help the patient overcome difficulties affecting therapy such as focus, concentration, or motivation. For example, “. . . and that psychologist has said, ‘well, I don’t think that we’re going to win because I think we need medication,’ Then I will listen to that psychologist” (P02).

### Drivers of Antidepressants

Participants reported a range of individual patient characteristics and demographics that shifted their decisions away from the default psychologist preference and toward other treatment options like antidepressants (see Figure 1). Participants described a systematic association between these cues and a psychologist referral, as each one gave them pause to consider more information as to whether a psychologist or antidepressants would be more effective. Individual factors included patients’ treatment preference, level of psychological insight, age, gender, past success or family history of success with antidepressants, and perceived lack of accessibility of a psychologist. A patient’s preference for antidepressants or against seeing a psychologist was associated with antidepressants and was a barrier to psychologist referrals, especially for male and older patients. Similarly, participants were more likely to prescribe antidepressants if a patient, or their blood relative, had previously made significant progress with antidepressants. For example, “. . . whether they’ve tried it in the past or whether they have a family member that’s tried a similar medication and helped them” (P05). subsequent prescription was common. Participants used insight to gauge the benefit a patient may receive from therapy, for example, “. . . (someone) who is pretty set in their ways and not terribly likely to change their habits and thinking, so maybe not respond so well to counselling at that age” (P12). Participants outlined a cluster of cues that caused them to consider shifting away from their heuristics and systematically assess which treatment would be more beneficial for the patient.

## Discussion

This study examined the decision cues that GPs use when deciding how to treat depressive symptoms and how they associate these cues with antidepressants and psychologist referrals. Contrary to previous literature, participants endorsed psychologist referrals over antidepressants when treating most depressive symptoms^26^ and described a key purpose of antidepressants as increasing functioning to engage with psychological therapy.^27^ Participants initiated antidepressants before psychologist referral only for severe depressive symptoms, including significant functional decline and suicidal ideation. The current Australian treatment guidelines for depression specify that psychological interventions alone are effective across the entire spectrum of depression severity and that antidepressants should be explored if symptoms do not improve with these interventions.^2^ The decision process outlined by participants appeared to be largely in line with current guidelines.^2^ Australian GPs have greater access to psychologists than many other Organisation for Economic Co-operation and Development countries, which has likely influenced their willingness to refer for depressive symptoms.^3^

One aspect of these results that deserves further consideration is how participants classified depressive symptom severity. Severe depressive symptoms were described as difficulty sleeping, anhedonia, loss of appetite, lack of motivation, and suicidal ideation. While these are indeed symptoms of MDD, a classification of “severe” is given when there is an excess of diagnostic symptoms and with marked impairment in social and occupational functioning.^28^ The depressive symptoms described by participants as severe, such as suicidal ideation, also align with mild or moderate MDD.^28^ Participants demonstrated an oversensitivity to depressive symptom severity and, by extension, to initiating antidepressant medications.

GPs are shown to have disparate assessments of depression severity compared with specialist contexts. Lampe et al.^12^ found that GPs have a more sensitive threshold for depression severity than the specialist diagnostic criteria used in treatment guidelines. GPs also demonstrate poor accuracy when diagnosing depression compared with symptom-screening questionnaires.^29^ Results from this article indicate GPs’ concept of severe depression is aligned with symptoms that only just meet the clinical threshold for diagnosing MDD. Australian GPs’ increased access to psychologist referrals has likely contributed to a broadening of their referral threshold, evidenced by the significant increase in psychologist referrals after the Better Access scheme was introduced.^3^

Previous investigation into antidepressant prescribing by GPs has uncovered a strong emphasis on patient risk and safety when treating depressive symptoms.^10^ This was the main exception to participants’ psychologist-default heuristic; however, suicidal ideation and functional decline are still encompassed by mild and moderate MDD, for which psychological therapy alone is the primary recommendation. In contrast with previous treatment guidelines,^30^ current guidelines specify that there is limited research indicating antidepressants are more effective than psychological interventions for moderate or severe MDD^2^ Current guidelines state, however, that antidepressants can still be appropriate as initial treatment, particularly for patients with previous success from antidepressants. GPs’ assessment of depression severity, and association of patient risk with antidepressants encompasses a broader range of depressive symptoms than outlined in treatment guidelines for prescribing antidepressants. Participants’ association between patient risk and antidepressants may account for some of the inconsistency between their responses and rising antidepressant prescriptions.

The mild to moderate symptoms that participants associated with psychologist referrals alone appeared to correspond with subclinical depressive symptoms. Primary mental health care encompasses a broader range of presentation severity than specialist mental health care does, including greater exposure to subclinical presentations.^12^ Treatment guidelines recommend subclinical depressive symptoms be treated initially with lifestyle interventions, guided self-help, and e-mental health programs and that patient preference (e.g., for psychologist referral) should be adhered to where possible.^2^ However, psychological therapy is a finite and expensive resource that is currently under significant strain, and subclinical depression can often be effectively treated with lifestyle interventions, e-mental health programs, or guided self-help.^31,32^ The large proportion of subclinical depression in primary care likely increases GPs’ sensitivity when assessing depressive symptom severity and increases the likelihood of psychologist referrals for subclinical depression.

Participants described a systematic decision process when treating patients who were older, male, had limited insight, had previous personal or family history of success with antidepressants, had restricted access to a psychologist, or had a preference for antidepressants. Any of these cues would cause GPs to stop and consider the patient’s symptoms and characteristics as a whole before deciding whether to recommend an antidepressant or a psychologist. Poor insight was associated with antidepressants and provided a barrier to psychologist referrals, aligning with evidence of a moderate correlation between patients’ insight into behaviour patterns and symptoms reduction from psychological therapy.^33^ It is still unclear, however, whether psychological therapy provides an added treatment benefit compared with antidepressants for patients with poor insight and also contrasts with findings that poor insight can hinder antidepressant effectiveness.^34^ Participants perceived older adults, males, and having low accessibility to a psychologist as reducing the likelihood of patient engagement with a psychologist. Accessibility is a strong barrier to engaging with psychological therapy, and males attend therapy at significantly lower rates than females do.^26^ Emerging evidence, however, contradicts the notion that older adults do not receive the same benefit from psychological therapy as middle-aged adults do. Adults older than 65 y often show more significant symptom improvement than middle-aged adults.^35^

### Implications of This Practice

Participants outlined a decision process for treating depressive symptoms in primary care that was largely aligned with treatment guidelines for depression. Their assessment of depressive symptom severity, however, and their associated thresholds for prescribing antidepressants and referring to psychologists appear to be overly sensitive when compared with treatment guidelines^2^ GPs in this study demonstrated discomfort for referring patients with suicidal ideation to a psychologist without initiating antidepressants, despite evidence showing psychological therapy as most effective in treating suicidal ideation.^36,37^ Antidepressants, however, are directly available for GPs to prescribe during a consult and are not subject to the same accessibility and waitlist challenges as referring to a psychologist.^10^

GPs should consider the range of patient symptom severity that they associate with psychologist referrals. It is very promising to witness participants’ openness and preference for psychologist referrals over antidepressant medication, considering past literature demonstrating the opposite. However, associations between psychologist referrals and subclinical symptoms suggests benefit may come from considering whether a greater proportion of patients whom GPs assess as mild to moderate can be treated without a psychologist and with more cost-effective means such as e-mental health programs, guided self-help, and lifestyle changes alone. In Australia and other countries, research and policy in treating depression is moving toward a stepped-care approach of using mental health resources in more efficient ways.^38,39^ The aim of stepped care for depression is to match presentations with treatments that balance efficacy with cost and availability of resources.

### Limitations and Future Directions

Results from this study were derived from participants’ recall of their decision processes, which may mean their responses were subject to recall bias or social desirability bias. Further, the findings may have been affected by self-selection bias and may represent decision-making processes used only by GPs interested in psychological research. These results may not be indicative of the broader GP population’s treatment decision processes for depressive symptoms nor GPs in countries and communities with less access to psychologists, and further research is warranted.

This research focused on antidepressants and psychologist referrals only and did not include e-mental health resources, GP counseling, or other treatments available to GPs for depressive symptoms. The omission of these additional treatment pathways may have elicited responses that do not encapsulate the extent of GPs’ decision processes. Future research should explore how GPs conceptualize and use these treatments and what types of depressive symptoms they are associated with.

## Conclusion

Many aspects of GPs’ decision framework are aligned with current literature and treatment guidelines for depression, except for prescribing antidepressants as the first treatment for mild and moderate MDD. Participants’ openness to psychologist referrals across the spectrum of severity is strongly supported by current treatment guidelines and contrasts with previous literature outlining GPs’ preference for antidepressants over psychological therapy. Consideration by GPs should be given to whether subclinical depressive symptoms can be effectively treated with cheaper and more accessible interventions before referring to a psychologist. The findings of the current article add to the literature showing that GPs not only prefer starting treatment for depressive symptoms with psychological therapy but also that GPs associate elevated patient risk with prescribing antidepressants.

## Supplemental Material

sj-docx-1-mdm-10.1177_0272989X231166009 – Supplemental material for A Qualitative Exploration of General Practitioners’ Treatment Decision-Making for Depressive Symptoms Click here for additional data file. Supplemental material, sj-docx-1-mdm-10.1177_0272989X231166009 for A Qualitative Exploration of General Practitioners’ Treatment Decision-Making for Depressive Symptoms by Alex Stallman, Nicola Sheeran and Mark Boschen in Medical Decision Making
