# Supplementary material for: A Qualitative Exploration of General Practitioners’ Treatment Decision-Making for Depressive Symptoms
Source: Med Decis Making. 2023 Apr 14;43(4):498–507. doi: 10.1177/0272989X231166009 (PMC10164610; doi:10.1177/0272989X231166009)
Supplement: sj-docx-1-mdm-10.1177_0272989X231166009 – Supplemental material for A Qualitative Exploration of General Practitioners’ Treatment Decision-Making for Depressive Symptoms [file sj-docx-1-mdm-10.1177_0272989X231166009.docx]

Appendix: Interview protocol

| *The purpose of this study is to find out specifically what GPs attend to while you’re figuring out how best to treat a patient’s depressive symptoms. I’m going to ask you to describe for me the patient information that you associate with different treatments for depressive symptoms. Some of these questions might feel repetitive, because I’m going to ask you about the same things in a few different ways, so don’t feel like you have to repeat yourself if you feel you’ve already answered something. As you answer, I just want you to think aloud, so verbalise any and all information that comes to mind related to the mental health treatment I ask you about. If you stop verbalising at any time when it looks like you’re thinking, I’ll just ask you to keep saying your thoughts.* | |
| --- | --- |
| *Question* | *Prompt* |
| 1. *What have you thought about when deciding whether or not a patient requires:* | - *anti-depressant medication for their depressive symptoms?* - *a referral to a Psychologist for their depressive symptoms?* - *you to manage their depressive symptoms yourself?* - *A combination of referral to a psychologist and anti-depressant medication for their depressive symptoms?* |
| 1. *What do you think contributes to whether a person will benefit from:* | - *antidepressant medication for depressive symptoms?* - *seeing a psychologist for depressive symptoms?* - *you managing their depressive symptoms yourself?* - *both seeing a psychologist and antidepressant medication for depressive symptoms?* |
| 1. *What type of person comes to mind when you think of someone who you:* | - *antidepressant medication for depressive symptoms?* - *seeing a psychologist for depressive symptoms?* - *you managing their depressive symptoms yourself?* - *both seeing a psychologist and antidepressant medication for depressive symptoms?* |
